# Functional Domain Order of an Anti-EGFR × Anti-CD16 Bispecific Diabody Involving NK Cell Activation

**DOI:** 10.3390/ijms21238914

**Published:** 2020-11-24

**Authors:** Atsushi Kuwahara, Keisuke Nagai, Takeshi Nakanishi, Izumi Kumagai, Ryutaro Asano

**Affiliations:** 1 Department of Biotechnology and Life Science, Graduate School of Engineering, Tokyo University of Agriculture and Technology, Tokyo 184-8588, Japan; s197158r@st.go.tuat.ac.jp (A.K.); kmiz@kuma.che.tohoku.ac.jp (I.K.); 2 Department of Biomolecular Engineering, Graduate School of Engineering, Tohoku University, Sendai 980-8579, Japan; nagai.keisuke@ma.mt-pharma.co.jp; 3 Department of Applied Chemistry and Bioengineering, Graduate School of Engineering, Osaka City University, Osaka 558-8585, Japan; nakanishi@osaka-cu.ac.jp

**Keywords:** bispecific diabody, cancer immunotherapy, CD16, EGFR, functional structure

## Abstract

Bispecific antibodies (bsAbs) have emerged as promising therapeutics. A bispecific diabody (bsDb) is a small bsAb consisting of two distinct chimeric single-chain components, with two possible arrangements of the domains. We previously reported the effect of domain order on the function of a humanized bsDb targeting the epidermal growth factor receptor (EGFR) on cancer cells, and CD3 on T cells. Notably, the co-localization of a T-cell receptor (TCR) with CD3 is bulky, potentially affecting the cross-linking ability of bsDbs, due to steric hindrance. Here, we constructed and evaluated humanized bsDbs, with different domain orders, targeting EGFR and CD16 on natural killer (NK) cells (hEx16-Dbs). We predicted minimal effects due to steric hindrance, as CD16 lacks accessory molecules. Interestingly, one domain arrangement displayed superior cytotoxicity in growth inhibition assays, despite similar cross-linking abilities for both domain orders tested. In hEx16-Dbs specifically, domain order might affect the agonistic activity of the anti-CD16 portion, which was supported by a cytokine production test, and likely contributed to the superiority of one of the hEx16-Dbs. Our results indicate that both the target antigen and mode of action of an antibody must be considered in the construction of highly functional bsAbs.

## 1. Introduction

Monoclonal antibodies have been widely used as therapeutics for various difficult-to-cure diseases, especially cancers and rheumatoid arthritis [1,2,3]. Although monoclonal therapeutic antibodies demonstrate considerable therapeutic benefits, due to their high specificity and binding affinity against target molecules, their application is restricted because of high production costs and requirements for a mammalian expression system. In addition, adverse clinical outcomes and data from animal studies have highlighted important limitations in their mode of action [3]. Many strategies have been explored to overcome these limitations. Bispecific antibodies (BsAbs), which have the ability to simultaneously bind two target molecules, represent one of the most attractive strategies. Although many studies have been conducted with bsAbs, blinatumomab (Blincyto^®^), designed to recruit T cells to the tumor site, is the only bsAb approved by the United States Food and Drug Administration for clinical use in cancer patients [4].

Over the last few decades, the construction of small bsAbs has also been developed using advanced recombinant technology. Some of the most successful small bsAb formats are diabodies (Dbs) [5], single-chain diabodies (scDbs) [6], tandem single-chain variable fragments (taFvs), such as blinatumomab [7], minibodies (dimeric scDb-CH3 fusion proteins) [8], and bispecific tetravalent antibodies (TandAbs) [9]. These small bsAbs are superior with respect to rapid tissue penetration and high target retention, in comparison to classic bsAbs prepared through chemical conjugation or quadroma production [10,11].

As demonstrated in the context of blinatumomab, cytotoxic T lymphocytes (CTLs) are often employed as the targets of bsAbs because of their strong cytotoxic activity against tumors or infected cells [4]. In addition to CTLs, natural killer (NK) cells, which are a component of the body’s innate immune system, have been targeted for antibody-based immunotherapy because of their potential to provide an early source of immunoregulatory cytokines and lyse target cells [12]. A variety of bsAbs that recruit NK cells have been reported [13]. Although NK cells have various activation pathways dependent on counter receptors, the pathway initiated through CD16A (FcγRIII) is one of the most effective, due to its involvement in antibody-dependent cell-mediated cytotoxicity (ADCC) [14]. However, the effects of Fc region-mediated ADCC were sometimes limited due to low affinities of the Fc region to CD16A and polymorphisms in CD16A influencing efficacy. Thus, anti-CD16A agonist antibodies have been focused and developed also integrating into several bsAb formats [15]. Indeed, the bispecific innate immune cell engagers, AFM13 [9] and AFM24 (Affimed, Heidelberg, Germany), targeting CD30 or EGFR on tumor cells and CD16A on NK cells, are currently in a phase I clinical study.

Bispecific Dbs (bsDbs) consist of two types of chimeric single-chain components, with each component having two possible domain orders, variable heavy domain–linker–variable light domain (VH-linker-VL) and VL-linker-VH. Thus, a bsDb has four possible domain orders. We have reported that the domain order affects the function of a humanized bsDb targeting EGFR on cancer cells and CD3 on T cells (hEx3-Dbs) [16]. In brief, the LH-type hEx3-Db (hEx3-LH), having both components in the VL-VH order, showed the highest cytotoxicity among the four domain arrangements possible. In the case of hE2x3-Db, in which the V region of the anti-EGFR antibody clone in hEx3-Db was replaced with that of another anti-EGFR clone, we also found that the LH type is the most functional format [17]. Furthermore, our comprehensive study revealed that there are similar tendencies in bsDbs with specificities for EGFR and CD3 targets, independent of the antibody clones used [18]. Flow cytometry studies revealed that the structure of the LH type may avert steric clashes with cell surface molecules, particularly those of T cells [16,17]. T-cell receptors co-localized with CD3 are relatively bulky [19,20], which might cause an increase in steric hindrance, affecting the cross-linking ability of a bsDb; however, whether this holds true in the case of bsDbs targeting other antigens is still unknown.

Here, we constructed and evaluated humanized bsDbs targeting EGFR and CD16 on NK cells (hEx16-Dbs) with four different domain orders. We speculated that any steric hindrance affecting the cross-linking abilities of the designed bsDbs would be minimalized, as there are no accessory molecules associated with CD16 [21]. Interestingly, unlike hEx3-Db, the HL-type hEx16-Db (hEx16-HL) inhibited cancer growth most effectively, although similar cross-linking abilities were predicted and observed for both hEx16-HL and the corresponding LH type (hEx16-LH). We speculate that, for hEx16-Dbs, domain order may affect the agonistic activity of an anti-CD16 antibody, thus contributing to the superiority of hEx16-HL. Our results indicated that the optimal domain order of a bsDb is dependent on both the target molecule and the mode of action of the antibody.

## 2. Results

### 2.1. Preparation of hEx16-Dbs with Four Different Domain Orders

**Figure 1:**
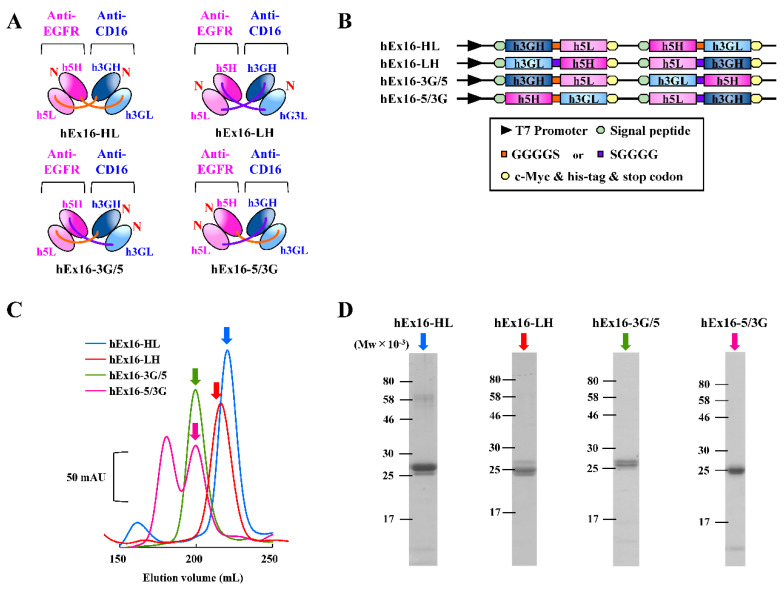
Preparation of hEx16-Dbs with different domain orders. (**A**) Schematic diagrams of four types of hEx16-Dbs: hEx16-HL, -LH, -3G/5, -5/3G. (**B**) Schematic diagrams of the co-expression vectors for four types of hEx16-Dbs. (**C**) Chromatographs of gel filtration of hEx16-Dbs and (**D**) SDS-PAGE analysis for dimer fractions indicated by arrows. mAU, milli-absorbance unit.

We have reported that the arrangement of domains within bsDbs affects their cancer growth inhibition efficiency [16,17]. A comprehensive investigation of bsDbs targeting EGFR and CD3 antigens revealed that the LH type induces the most effective anticancer response [18]; however, it is still unknown whether other bsDbs of the LH type also represent the most effective format. To further investigate the effect of domain order on the function of bsDbs, we prepared hEx16-Dbs with four possible domain orders using an *Escherichia coli* expression system. Schematic diagrams and gene constructs are summarized in Figure 1A,B. The his-tag purified hEx16-Dbs, extracted from the culture supernatant and bacterial soluble fractions, were subjected to gel filtration analysis to fractionate the dimers of each hEx16-Db. A single peak was observed in the resulting chromatograms for each construct, except for hEx16-5/3G, (Figure 1C), and the purity of the fractionated dimers was confirmed by sodium dodecyl sulfate polyacrylamide gel electrophoresis (SDS-PAGE) analysis under reducing conditions (Figure 1D and Figure S1, Supplementary Materials). The final yields of hEx16-HL, hEx16-LH, hEx16-3G/5, and hEx16-5/3G were 0.43, 0.59, 0.51, and 0.36 mg/L, respectively. Taken together, these results demonstrate the successful preparation of hEx16-Dbs.

### 2.2. Effect of the Domain Order of hEx16-Dbs on Growth Inhibition

**Figure 2:**
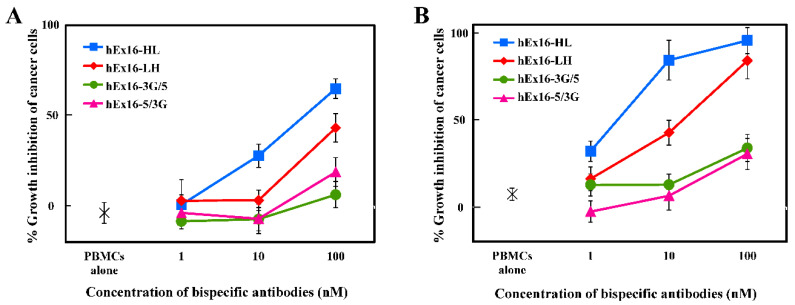
Growth inhibition of EGFR-positive TFK-1 cells by hEx16-Dbs. The purified hEx16-Dbs were added with peripheral blood mononuclear cells (PBMCs) to TFK-1 cells and growth inhibition was evaluated using the MTS assay. The ratio of TFK-1:PBMC was either (**A**) 1:30 or (**B**) 1:80.

To evaluate the influence of domain order in hEx16-Dbs on the growth inhibition of human carcinoma (TFK-1) cells, we analyzed four types of fractionated hEx16-Db dimers using peripheral blood mononuclear cells (PBMCs), as effector cells. In contrast to hEx3-Dbs, the highest growth inhibitory effect was observed for the HL-type hEx16-Db (Figure 2A,B). The LH type, while weaker than the HL type, displayed a higher growth inhibitory effect than both the 3G/5 and 5/3G types. This growth inhibitory tendency differs from that observed in hEx3-Dbs, where the weakest effects were observed for the HL-type domain arrangement [16,17]. These results suggest that the functional domain order yielding the maximum efficacy depends on the antibodies involved and/or the antigen targeted.

### 2.3. Preparation of hEx16-scDbs with Different Domain Orders

**Figure 3:**
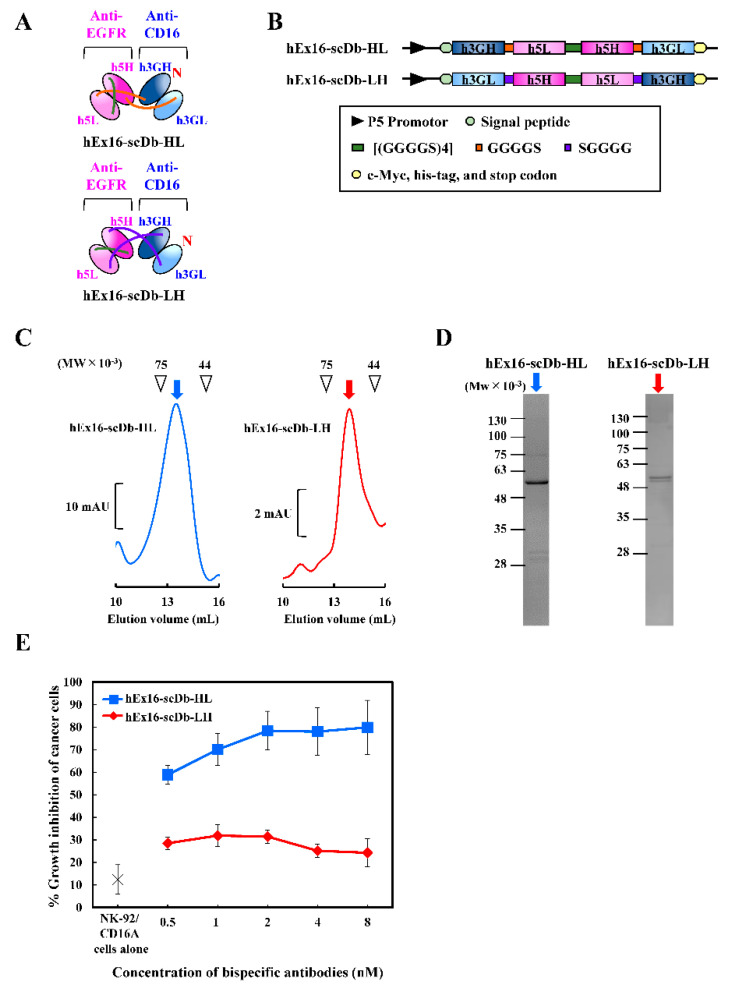
Preparation and evaluation of hEx16-scDbs with different domain orders. (**A**) Schematic diagrams of the two types of hEx16-scDbs: hEx16-scDb-HL and -LH. (**B**) Schematic diagrams of the vectors designed for the two types of hEx16-scDbs. (**C**) Gel filtration chromatographs of hEx16-scDbs and (**D**) SDS-PAGE analysis of monomer fractions indicated by arrows. mAU, milli-absorbance unit. (**E**) Growth inhibition of EGFR-positive TFK-1 cells by hEx16-scDbs. The purified hEx16-scDbs were added with the NK-92/CD16A cell line to TFK-1 cells. The ratio of TFK-1:NK-92/CD16A was 4:1.

In the preparation of Dbs using co-expression vectors, it is often difficult to produce purely homogeneous heterodimers. Expression levels of each chimeric single-chain component cannot be regulated equally, which leads to the formation of inactive homodimers that are not easily removed using gel filtration chromatography. To prepare homogeneous small bsAbs, we constructed HL- and LH-type hEx16-scDbs in which the chimeric single-chain components were connected using an additional middle linker (Figure 3A,B). We first expressed hEx16-scDbs using an *E. coli* system. To evaluate the effect of host cell impurities and effector cell dependency, we performed an MTS assay using the culture supernatant directly. A higher growth inhibitory effect was again observed using HL-type hEx16-Db (Figure S2A), and no growth inhibition was observed when we used CD16-negative lymphokine-activated killer cells with the T-cell phenotype, as the effector cell (Figure S2B), indicating that impurities have no cytotoxic activity and hEx16-scDb function is dependent on the effector cells. However, we could not isolate and purify soluble hEx16-scDbs for further investigation by using an *E. coli* expression system. We then used the *Brevibacillus choshinensis* expression system, which offers several advantages [22,23,24], as demonstrated in our recent preparation of small bispecific antibodies, including hEx3-scDbs [25]. In the present study, we confirmed the successful preparation of soluble hEx16-scDbs using *B. choshinensis*, followed by gel filtration chromatography and SDS-PAGE analysis under reducing conditions (Figure 3C,D and Figure S3A,B). The final yields of hEx16-scDb-HL and hEx16-scDb-LH were 0.64 and 0.073 mg/L, respectively.

### 2.4. Effect of the Domain Order of hEx16-scDbs on Growth Inhibition

PBMCs are composed of several types of immune cells, such as T cells and B cells, creating donor-dependent composition differences and concerns with regard to donor-to-donor variability. In addition to the scDb format, we used a CD16-expressing NK-92/CD16A cell line, as the effector cell, to evaluate the influence of domain order on growth inhibition. In contrast to the results obtained using PBMCs, hEx16-scDbs inhibited cancer growth at a low Effector/Target cells ratio and, consistent with the hEx16-Dbs, the HL-type domain arrangement displayed higher growth inhibitory effects in comparison to the LH type (Figure 3E). To verify target antigen specificity, we performed the MTS assay using hEx16-scDb-HL with either cetuximab, 528 Fab, or 3G8 IgG (Figure S4A,B). When cetuximab was co-cultured with hEx16-scDb-HL, the expected additive effect was observed, in comparison to hEx16-scDb-HL alone, as cetuximab can cross-link with the target cells used. In contrast, co-culturing hEx16-scDb-HL with 528 Fab or 3G8 IgG resulted in a decrease in cytotoxicity, in comparison to hEx16-scDb-HL alone, due to inhibition of hEx16-scDb-HL cross-linking by competitive binding of 528 Fab to EGFR or 3G8 IgG to CD16, respectively. These results confirm the target specificity of the HL-type domain arrangement, and establish it as the superior format in bsDbs targeting EGFR and CD16.

### 2.5. Binding Ability of Each hEx16-Db

**Figure 4:**
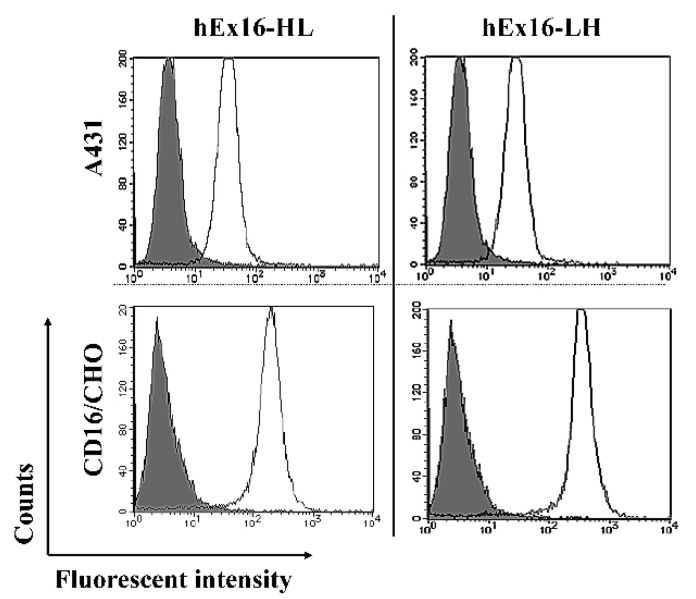
Evaluation of the cross-linking abilities of the hEx16-Dbs. EGFR-positive A431 cells and CD16/CHO cells were incubated with PBS, as a negative control (shaded area), or with each hEx16-Db (open area), followed by staining with fluorescein isothiocyanate (FITC)-sCD16 for A431 cells (upper panels) or with FITC-sEGFR for CD16/CHO cells (lower panels).

Previously, we speculated that the structure of the LH-type hEx3-Db was capable of avoiding steric hindrance with cell surface molecules, and this might contribute to its superior cancer growth inhibition effect, independent of target affinity. To extend this evaluation to the hEx16-bsDbs, we first evaluated the binding affinities to each antigen using surface plasmon resonance (SPR) spectroscopy. The results revealed no measurable differences in binding affinity among the hEx16-bsDb types, with the exception of hEx16-3G/5 and hEx16-5/3G, targeting the sEGFR and sCD16 antigens, respectively (Table 1 and Table 2, Figures S5 and S6). Subsequently, we evaluated the cross-linking abilities of the bsDbs using flow cytometry. Both hEx16-HL and hEx16-LH showed similar cross-linking abilities for both EGFR-positive A431 cells with fluorescein isothiocyanate (FITC)-labeled sCD16, and CD16-expressing Chinese hamster ovary (CD16/CHO) cells with FITC-labeled sEGFR (Figure 4). These results suggest that there are no differences in the steric hindrance experienced by the hEx16-Dbs, and thus cross-linking ability is not a key factor contributing to the superior growth inhibition observed for hEx16-HL.

### 2.6. Cytokine Release Induced by hEx16-Db Binding

**Figure 5:**
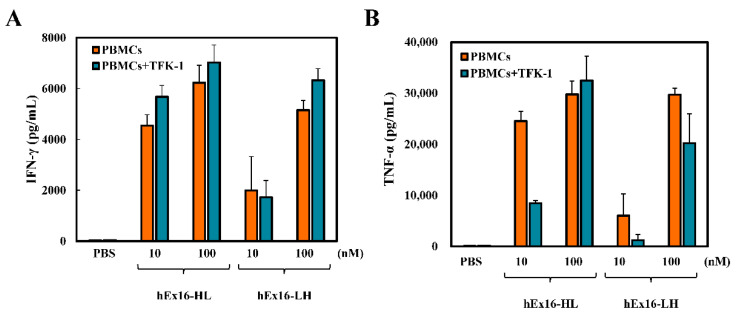
hEx16-Dbs-mediated cytokine production by PBMCs. PBMCs were cultured with hEx16-Dbs, either in the presence or absence of TFK-1 cells. Concentrations of (**A**) IFN-γ and (**B**) TNF-α were evaluated using enzyme-linked immunosorbent assays.

In our experience, bsDbs targeting EGFR and CD3 with superior growth inhibition for LH-type domain arrangements also induce higher levels of cytokine release [16,17]. To investigate factors contributing to the enhanced cytotoxicity observed, we next evaluated interferon (IFN)-γ and tumor necrosis factor (TNF)-α production from CD16 positive cells, in PBMCs, mediated by the hEx16-Dbs. Both TNF-α and IFN-γ are known to contribute to cancer growth inhibition. The analysis was conducted by measuring the concentration of each cytokine in the supernatant of PBMCs cultured with a hEx16-Db construct, either in the presence or absence of TFK-1 cells. In comparison to hEx16-LH, hEx16-HL induced higher levels of cytokine release (Figure 5). The presence of TFK-1 cells did not affect cytokine release considerably, and was found to reduce the production of TNF-α at 10 nM. Taken together, our results indicate that cytokine production is responsible for the differences in growth inhibition observed for the distinct domain arrangements of hEx16-Dbs.

## 3. Discussion

We have reported that rearranging the domain order affected the cancer growth inhibitory activity of humanized bsDb targeting EGFR and CD3 [16]. The highest inhibitory effect was observed for the LH type of hEx3-Dbs, and similar tendencies were confirmed with other bsDbs targeting EGFR and CD3 [17,18,25]. CD3 co-localizes with a relatively bulky TCR, and a recent study using cryo-electron microscopy clearly revealed the large, fully assembled structure of CD3 and TCR [20]. Supported by flow cytometric analysis [16,17], we speculated that rearranging the domain order of bsDbs resulted in differences in the steric hindrance experienced upon TCR binding to CD3, affecting the cancer growth inhibitory activity of the bsDbs.

CD16 expressed on NK cells is composed of two immunoglobulin fold domains, and, unlike CD3, there are no related accessory molecules [21,26]. Thus, the cross-linking ability of a bsDb targeting CD16 may not be affected by steric hindrance, and thus rearranging the domain order may not affect function. To verify this hypothesis, we constructed bsDbs targeting EGFR and CD16 with four different domain orders and two types of single chains to prevent dissociation of the chimeric single-chain components and ensure the preparation of homogenous bsDbs. However, increasing the molecular size of the construct, in this case by including a linker, often leads to difficulties in production using a simple *E. coli* expression system. We recently reported the utility of the *B. choshinensis* expression system for the preparation of scDbs [25]. This nonpathogenic Gram-positive bacterium can directly secrete recombinant proteins into culture media without concerns related to contaminating endotoxins [22,23,24]. In this study, we successfully prepared hEx16-Dbs and -scDbs by using *E. coli* (Figure 1C,D) and *B. choshinensis* expression systems (Figure 3C,D), respectively.

Growth inhibition assays revealed that hEx16-HL was the most efficient cytotoxic agent among the four hEx16-Dbs (Figure 2), unlike our previous result with bsDbs targeting EGFR and CD3 [16,17,18]. In the scDb format, hEx16-scDb-HL also showed higher activity than the corresponding LH type (Figure 3E). The EC_50_ of hEx16-scDb-HL was 0.30 nM, which is similar to our previous report on the cytotoxicity of hEx16-HL with an approved therapeutic anti-EGFR antibody, cetuximab [27]. A reduction in the affinity of the anti-EGFR antibody 528, used in hEx16-Dbs, was observed after humanization [28]. We also isolated high-affinity humanized 528 VH mutants by using a phage display method and observed increased cancer growth inhibition upon integration of these mutants into hEx3-Dbs [29,30,31]. Thus, integration of these mutants into hEx16-Dbs would be expected to further increase cancer growth inhibition. SPR imaging revealed that there were no measurable differences in the binding affinity between the HL and LH types (Table 1 and Table 2). Of note, hEx16-3G/5 and hEx16-5/3G, which are considered parallel types having their chimeric single-chain components mixed with respect to the VH–VL and VL–VH order [17], showed reduced binding affinity to EGFR and CD16, respectively (Table 1). This phenomenon was previously observed with bsDbs targeting EGFR and CD3 [16,17], where structural modeling revealed that the N-terminal side of the Fv in parallel types partially overlaps the paratope surface on the C-terminal side of the Fv and impedes binding [17]. In contrast to binding, it is difficult to estimate the cytotoxic efficiency of parallel types; for example, parallel types of E2x3-Dbs were less cytotoxic than their anti-parallel types (both chimeric single-chain components are VH–VL or VL–VH order) [17] like hEx16-Dbs, and effects of parallel types of hEx3-Db ranked between LH and HL types [16]. Structural changes may occur after accessing and binding of parallel diabodies to target cells, but such phenomena are unpredictable.

**Figure 6:**
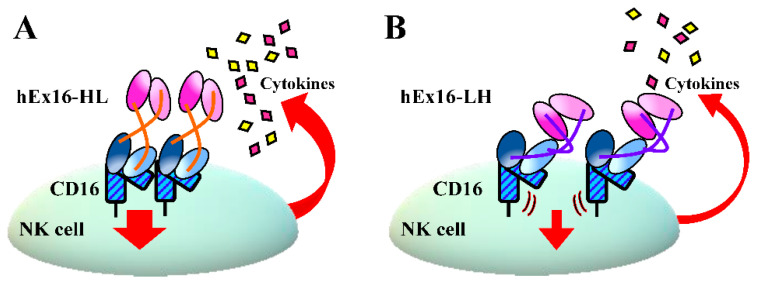
A proposed model for the differences in the activation of NK cells mediated by hEx16-Dbs binding. (**A**) The binding of hEx16-HL does not inhibit CD16 dimerization and facilitates induction of intracellular signaling. (**B**) The binding of hEx16-LH causes steric hindrance in CD16 dimerization and weakens the intracellular signaling.

Consistent with our expectation, and unlike Dbs targeting EGFR and CD3 [16,17], flow cytometric analysis revealed similar cross-linking abilities of hEx16-HL and hEx16-LH for target cell lines and FITC-labeled target antigens (Figure 4). In our previous work, the cytotoxicity of bsDbs targeting EGFR and CD3 was associated with cytokine production by effector cells [16,17], and the presence of target cells increased the effect substantially. In contrast, while the cytotoxicity of hEx16-Dbs was also correlated with cytokine production, the effect was decreased in the presence of target cells (Figure 5). These results imply that rearrangement of the domain order in hEx16-Dbs affects cytotoxicity differently than bsDbs targeting EGFR and CD3. Dimerization of CD16 is a key step in the signal transduction pathway of NK cell activation [8,32], and the parental anti-CD16 antibody clone 3G8, used in hEx16-Dbs, is a known agonist for NK cells [33]. Upon binding to CD16, the configuration of hEx16-LH may inhibit the dimerization of CD16, which could account for the differences observed in cytokine production and cytotoxicity of hEx16-Dbs (Figure 6).

In conclusion, we constructed and evaluated domain-rearranged hEx16-Dbs targeting EGFR and CD16, to investigate whether steric hindrance with the associated TCR affects the function of bsDbs. We observed similar cross-linking abilities for all hEx16-Dbs, as per our expectation, since CD16 has no related accessory molecules. However, we did observe significant differences in the cytotoxicity of the hEx16-Dbs, which might be due to an agonistic activity on effector cells. Another study recently reported that the distance between target antigens can have an impact on the function of bsAbs, which is another possible reason for our observations [34]. Taken together, our results indicate that the optimal domain order of bsDbs is dependent on both the target molecules and mode of action of the antibody, and these factors must be taken into consideration for the construction of highly functional bsAbs.

## 4. Materials and Methods

### 4.1. Construction of Expression Vectors for hEx16-Dbs and hEx16-scDbs

We previously designated the VH and VL regions of the humanized anti-EGFR antibody 528 as h5H and h5L [35], and the humanized anti-CD16 antibody 3G8 as h3GH and h3GL [27], as well as reported the construction of bacterial co-expression vectors for hEx3-Dbs with different domain orders [16]. Following the same procedure as for hEx3-Dbs, we constructed bacterial co-expression vectors for hEx16-Dbs with the following four different domain orders: pRA-hEx16-HL for hEx16-HL, in which both chimeric single-chain components are in the VH–VL order; pRA-hEx16-LH for hEx16-LH, in which both chimeric single-chain components are in the VL–VH order; pRA-hEx16-3G/5 for hEx16-3G/5, in which both the V regions of the humanized anti-CD16 antibody 3G8 are located at the N-terminus; and pRA-hEx3-5/3G for hEx3-5/3G, in which both the V regions of the humanized anti-EGFR antibody 528 are located at the N-terminus.

We previously constructed pROXb3-hEx3-scDb vectors for the expression of hEx3-scDbs in *B. choshinensis* [25]. Both genes for hEx16-scDb-HL and hEx16-scDb-LH were amplified by overlapping polymerase chain reactions (PCRs), and then inserted into the pROXb3 expression vector to construct pROXb3-hEx16-scDb-HL for hEx16-scDb-HL and pROXb3-hEx16-scDb-LH for hEx16-scDb-LH, using restriction enzymes.

### 4.2. Preparation of hEx16-Dbs and hEx16-scDbs

The four hEx16-Dbs representing the four different possibilities of domain order were prepared using an *E. coli* expression system, as described previously [16]. Briefly, *E. coli* strain BL21 Star (DE3) was transformed with each pRA-hEx16-Db vector individually and the resulting hEx16-Db protein was purified using immobilized metal affinity chromatography (IMAC) directly from the bacterial supernatant and periplasmic fractions. Size exclusion chromatography, using either a Hiload 26/60 Superdex 200-pg or Superdex 200 Increase 10/300 GL column (GE Healthcare Bio-Science, Piscataway, NJ, USA), was used to fractionate the dimers of each hEx16-Db. Briefly, the column was first equilibrated with phosphate buffered saline (PBS), and then each his-tag purified hEx16-Db was loaded onto the column at a flow rate of 2.0 mL/min. The purity was confirmed by SDS-PAGE under reducing conditions.

The two hEx16-scDbs were prepared using the *B. choshinensis* expression system, as previously reported [25], with the exception of 1% Phytone^TM^ peptone (BD Biosciences, San Jose, CA, USA) instead of 4%. After purification of each hEx16-scDb through IMAC and gel filtration, the purity of each fractionated hEx16-scDb was confirmed by SDS-PAGE under reducing conditions.

### 4.3. In Vitro Growth Inhibition Assay

A human bile duct carcinoma (TFK-1) highly expressing EGFR cell line, established in our laboratory [36,37], was used as the target cancer cell for this study. We used PBMCs or NK92/CD16A cell line as effectors [38] to evaluate the growth inhibition activity of hEx16-Dbs or hEx16-scDbs, respectively. Authorized PBMCs were purchased from Cellular Technology Limited (Cleveland, OH, USA). To confirm target antigen specific growth inhibition, cetuximab (Bristol Myers Squibb, New York, NY, USA) and FITC-3G8 IgG (Adipogen Life Sciences, San Diego, CA, USA) were used. 528 Fab was prepared through papain digestion using the Pierce™ Fab Preparation Kit (Thermo Fisher Scientific, Waltham, MA, USA). The in vitro inhibition of cancer cell growth was evaluated using a 3-(4,5-dimethylthiazole-2-yl)-5-(3-carboxymethoxyphenyl)-2-(4-sulfophenyl)-2*H*-tetrazolium inner salt (MTS) assay kit (CellTiter 96 AQueous Non-Radioactive Cell Proliferation Assay; Promega, Madison, WI, USA) as reported previously [35].

### 4.4. Surface Plasmon Resonance Spectroscopy

The binding of hEx16-Dbs and -scDbs against soluble EGFR (sEGFR) and soluble CD16 (sCD16) was evaluated using SPR spectroscopy (Biacore 2000 or Biacore T-200, GE Healthcare). The methods for the expression and purification of sEGFR have been described previously [28]. sCD16 used for the evaluation of hEx16-Dbs was prepared in accordance with a previously reported method [39]. Briefly, the preparation of sCD16 involved Expi293F cells transformed with a pCAGGS-sCD16 [40] vector by transfection, followed by purification of the culture supernatant using IMAC.

sEGFR and sCD16 for hEx16-Dbs binding assays were immobilized on cells in a CM5 sensor chip up to 1554 and 1590 resonance units, respectively, while sEGFR and sCD16 for hEx16-scDbs binding assays were immobilized in a similar fashion, up to 2289 and 2416 resonance units, respectively. Various concentrations of hEx16-Dbs or hEx16-scDbs in PBS containing 0.005% Tween 20 were allowed to flow over each bound antigen. The association and dissociation used times were 120 s each. The surface was regenerated with 10 mM glycine-HCl (pH 2.0), with no loss of activity. The data were referenced by subtracting the response of a blocked blank cell. BIAevaluation software (GE Healthcare) was used to analyze the data. Kinetic parameters were calculated by a global fitting analysis with the assumption of a 1:1 Langmuir binding model.

### 4.5. Confirmation of Cross-Linking Ability

Fluorescein isothiocyanate-labeled sCD16 (FITC-sCD16) and sEGFR (FITC-sEGFR) were prepared using the Fluorescein Labeling Kit-NH2 (Dojindo Laboratories, Kumamoto, Japan) to confirm the cross-linking efficiency between sCD16 and EGFR-positive A431 cells and sEGFR and CD16/CHO cells [27]. Approximately 1 × 10^6^ target cells were incubated for 30 min on ice with 200 pmoles of hEx16-HL or hEx16-LH. After washing with PBS, the cells were exposed for 30 min to 1 µg of FITC-sCD16 or FITC-sEGFR on ice. Stained cells were subsequently analyzed by flow cytometry (FACSCalibur, Becton Dickinson, San Jose, CA, USA).

### 4.6. Enzyme-Linked Immunosorbent Assay

Each hEx16-Db, at a final concentration of 10 or 100 nM, was co-cultured with PBMCs (4.0 × 10^5^ cells), in the presence or absence of overnight-adhered TFK-1 cells (5.0 × 10^3^ cells), in 96-well plates. After 24 h, supernatants were harvested and used in enzyme-linked immunosorbent assays (ELISA), for IFN-γ and TNF-α, according to the manufacturer’s instructions (ELISA Ready-SET-Go!, Bay Bioscience, Hyogo, Japan).

### 4.7. Cell Lines

Human bile duct carcinoma (TFK-1), human epidermoid cancer (A431), CD16/CHO, and NK-92/CD16A cell lines were used in this study. These cell lines were cultured in RPMI 1640 medium supplemented with 10% fetal bovine serum, 100 U/mL penicillin, and 100 µg/mL streptomycin. The NK-92/CD16A cell line was kindly provided by Professor Tachibana of Osaka City University, Japan, and cultured in MyeloCult H5100 medium (StemCell Technologies, Vancouver, BC, Canada) supplemented with 100 U/mL of recombinant human interleukin-2 (Imunace, Shionogi, Osaka, Japan).

## Figures and Tables

**Table 1:** Binding parameters of hEx16-Dbs obtained using surface plasmon resonance (SPR) spectroscopy.

	sEGFR			sCD16		
	*k*_on_ (×10^4^ M^−1^s^−1^)	*k*_off_ (×10^−2^ s^−1^)	*K*_D_ (×10^−8^ M)	*k*_on_ (×10^4^ M^−1^s^−1^)	*k*_off_ (×10^−2^ s^−1^)	*K*_D_ (×10^−8^ M)
hEx16-HL	7.44	0.637	8.57	2.45	1.45	59.1
hEx16-LH	6.66	0.877	13.2	5.07	2.69	53.2
hEx16-3G/5	0.000742	0.00106	143	4.33	2.29	52.8
hEx16-5/3G	3.76	0.801	21.3	0.0073	2.24	30,600

**Table 2:** Binding parameters of hEx16-scDbs obtained using surface plasmon resonance spectroscopy.

	sEGFR			sCD16		
	*k*_on_ (×10^4^ M^−1^s^−1^)	*k*_off_ (×10^−2^ s^−1^)	*K*_D_ (×10^−8^ M)	*k*_on_ (×10^4^ M^−1^s^−1^)	*k*_off_ (×10^−2^ s^−1^)	*K*_D_ (×10^−8^ M)
hEx16-scDb-HL	18.0	0.470	2.61	10.5	2.59	24.7
hEx16-scDb-LH	10.6	0.170	1.60	2.77	0.579	20.9

## Supplementary Materials

The following are available online at https://www.mdpi.com/1422-0067/21/23/8914/s1.

## Abbreviations

bsAbs	bispecific antibodies
bsDbs	bispecific diabodies
VH	variable heavy domain
VL	variable light domain
EGFR	epidermal growth factor receptor
TCR	T-cell receptor
NK	natural killer
Dbs	diabodies
scDbs	single-chain diabodies
taFvs	tandem single-chain variable fragments
CTLs	cytotoxic T lymphocytes
ADCC	antibody-dependent cell-mediated cytotoxicity
SDS-PAGE	sodium dodecyl sulfate polyacrylamide gel electrophoresis
PBMCs	peripheral blood mononuclear cells
IMAC	immobilized metal affinity chromatography
PBS	phosphate buffered saline
MTS	3-(4,5-dimethylthiazole-2-yl)-5-(3-carboxymethoxyphenyl)-2-(4-sulfophenyl)-2*H*-tetrazolium inner salt
sEGFR	soluble EGFR
sCD16	soluble CD16
ELISA	enzyme-linked immunosorbent assays
TNF	tumor necrosis factor
IFN	interferon
