# Supplementary material for: Functional Domain Order of an Anti-EGFR × Anti-CD16 Bispecific Diabody Involving NK Cell Activation
Source: Int J Mol Sci. 2020 Nov 24;21(23):8914. doi: 10.3390/ijms21238914 (PMC7727810; doi:10.3390/ijms21238914)
Supplement: Supplementary file 1 [file ijms-21-08914-s001.pdf]

Figure S1

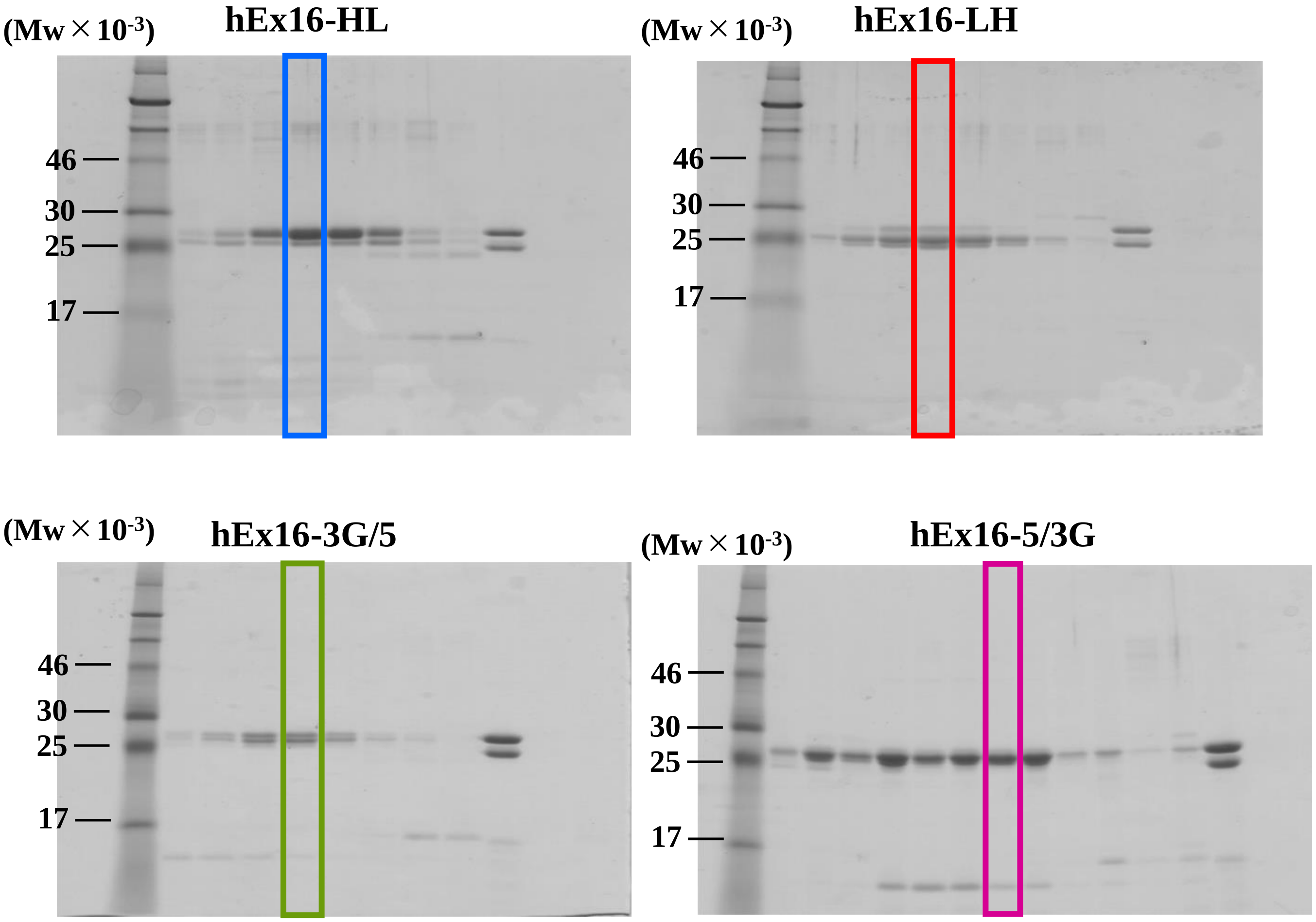

Full sodium dodecyl sulfate polyacrylamide gel electrophoresis (SDS-PAGE) image corresponding to Fig. 1D. The purity of hEx16-HL, hEx16-LH, hEx16-3G/5, and hEx16-5/3G calculated from band intensities were 85%, 100%, 95%, and 96%, respectively.

Figure S2

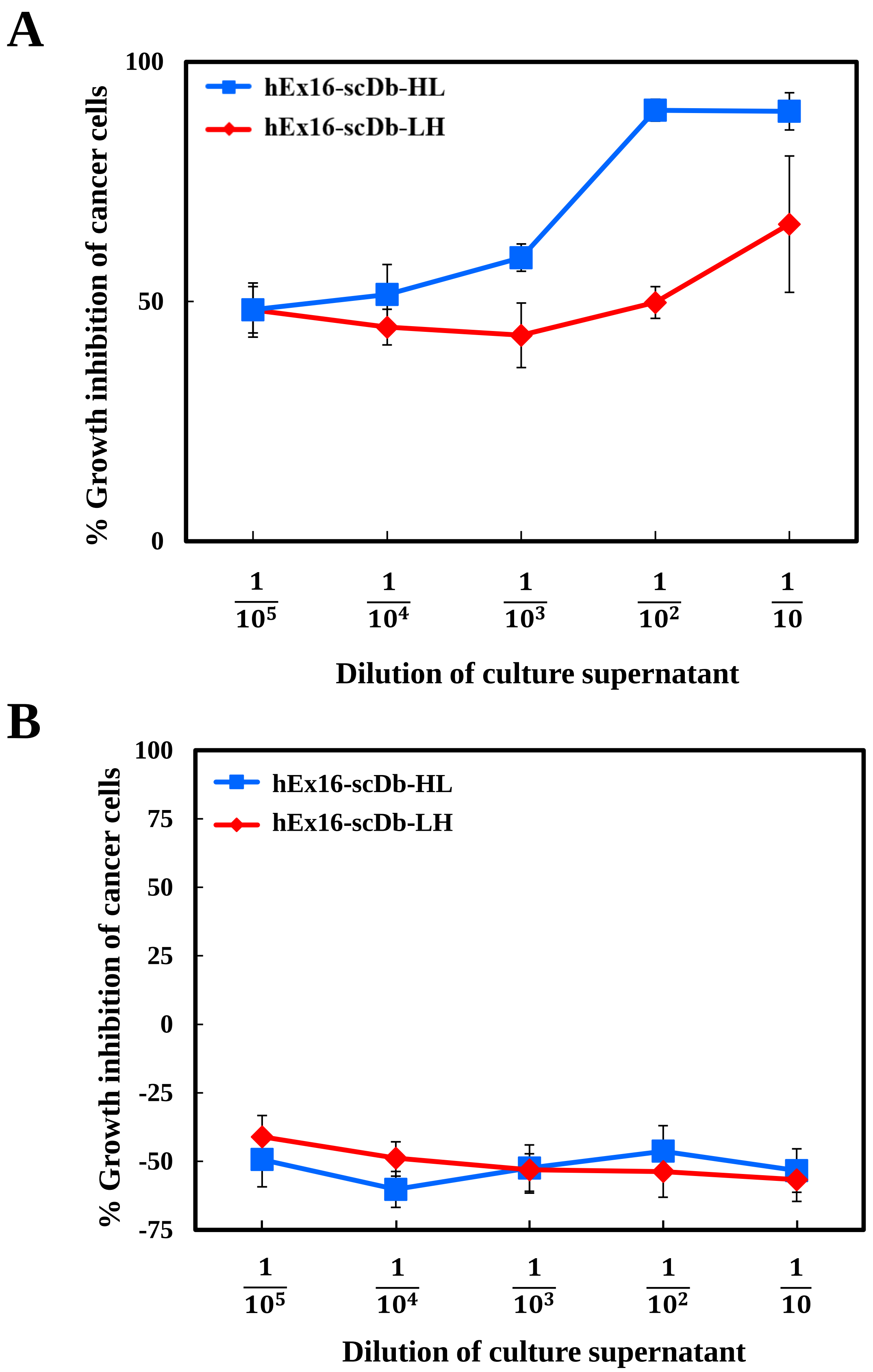

Effect of host cell impurities and effector cell dependency. hEx16-scDb-HL and -LH were expressed using *Escherichia coli* strain BL21 Star (DE3). Diluted culture supernatants and NK-92/CD16A cell (A) or lymphokine-activated killer cells with the T-cell phenotype (B) were added to TFK-1 cells. The ratios of TFK-1: effector cells were 4:1.

Figure S3

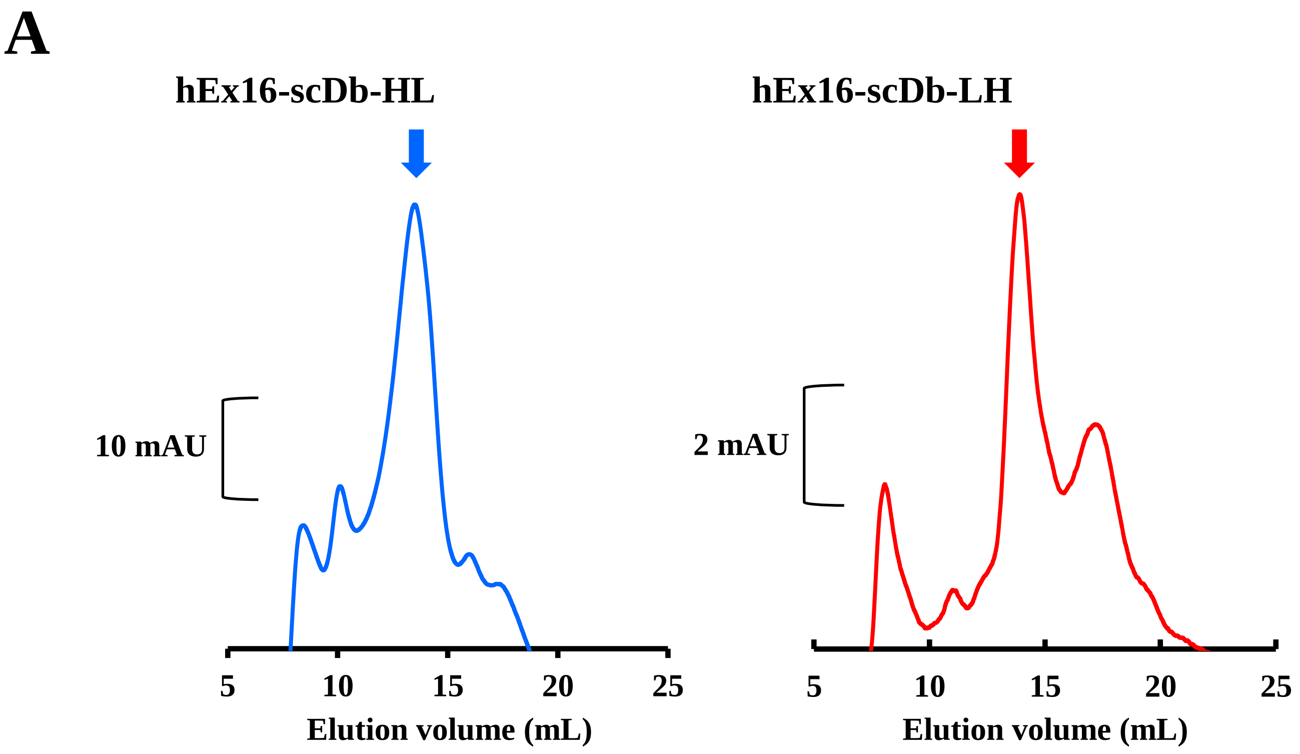

Full analytical size exclusion chromatograph of hEx16-scDbs corresponding to Figure 3C**.** The calculated peak area of monomer fraction for hEx16-scDb-HL and hEx16-scDb-LH were 50% and 45%, respectively.

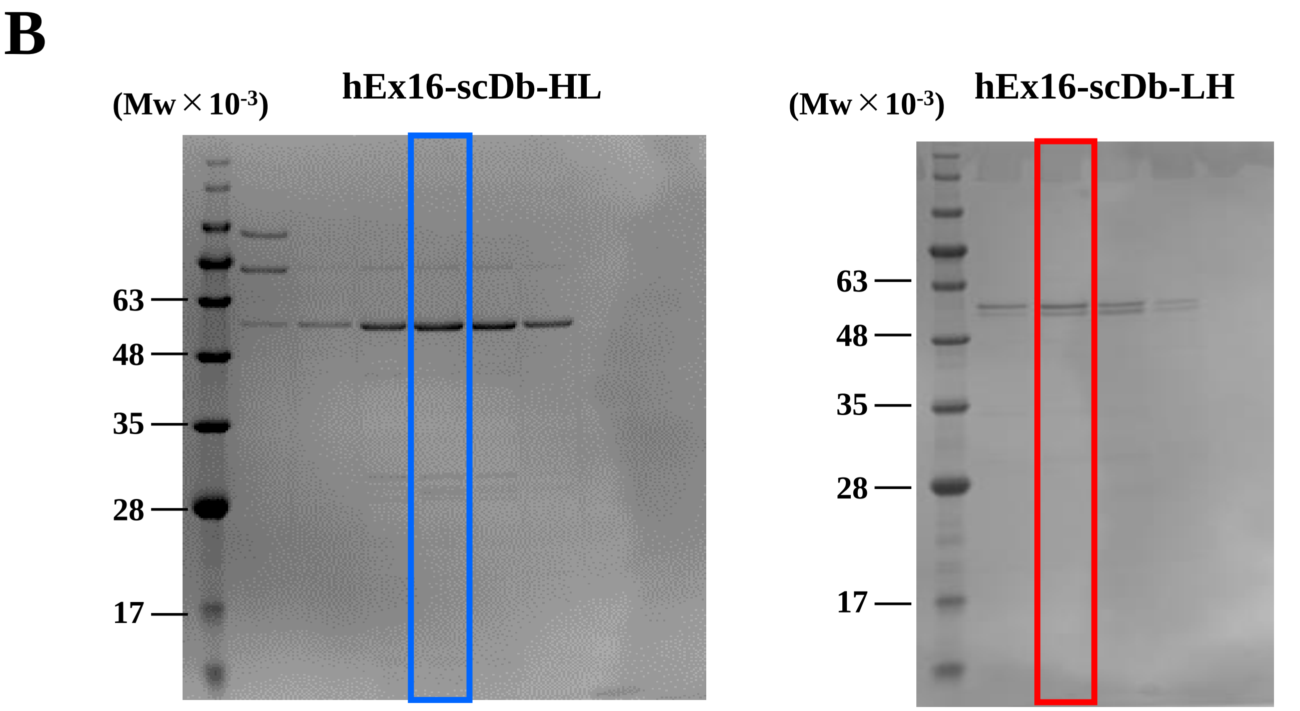

Full sodium dodecyl sulfate polyacrylamide gel electrophoresis (SDS-PAGE) image corresponding to Fig. 3D. The purity of hEx16-scDb-HL and hEx16-scDb-LH calculated from band intensities were 98%, and 95%, respectively.

Figure S4

A

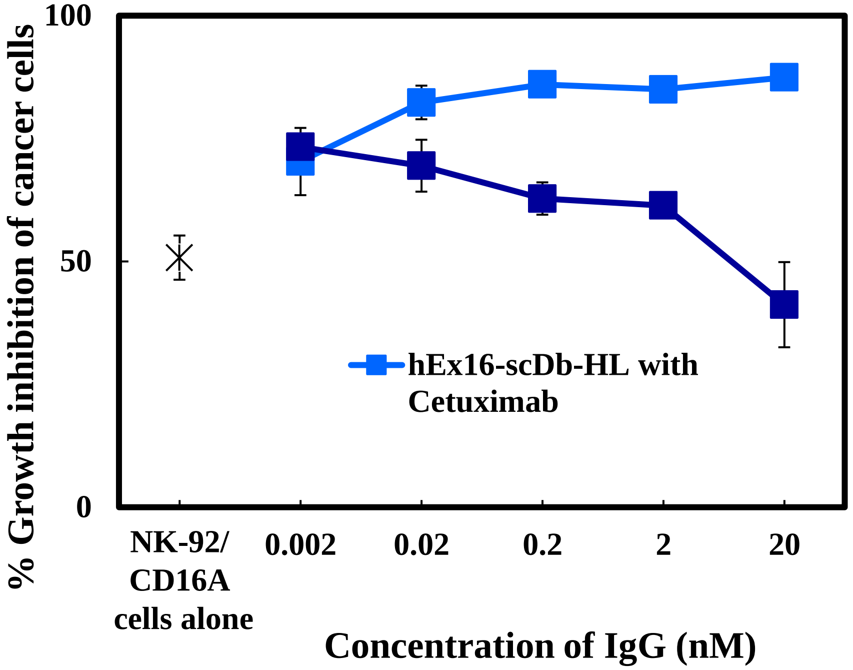

Confirmation of target antigen specific growth inhibition effects. Two nM concentration of hEx16-scDb-HLs with Cetuximab or anti-CD16 3G8 IgG were added with NK-92/CD16A cell line to TFK-1 cells. The ratio of TFK-1: NK-92/CD16A was 10:1.

B

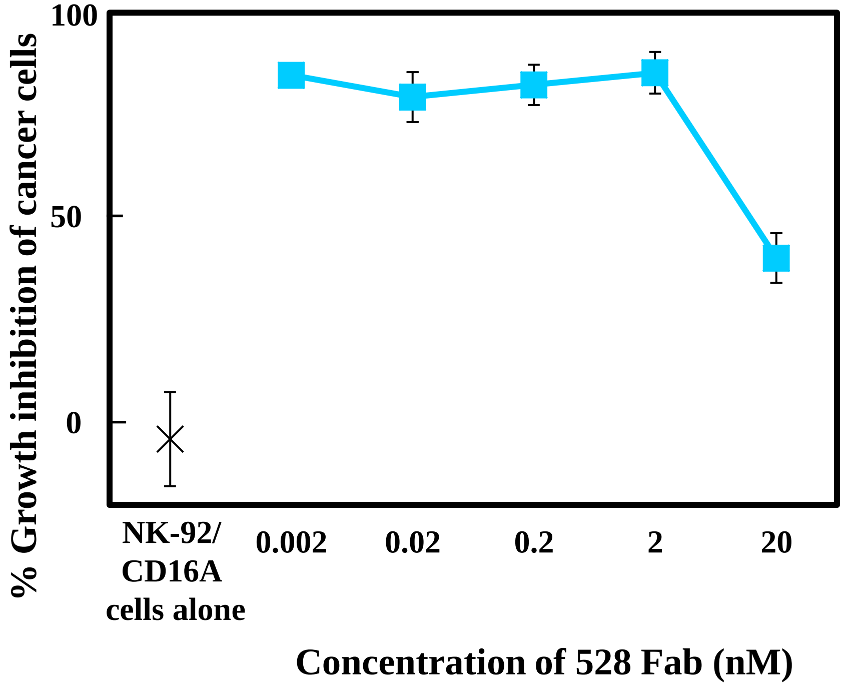

Confirmation of target antigen specific growth inhibition effects. Two nM concentration of hEx16-scDb-HLs with 528 Fab were added with NK-92/CD16A cell line to TFK-1 cells. The ratio of TFK-1: NK-92/CD16A was 4:1.

Figure S5

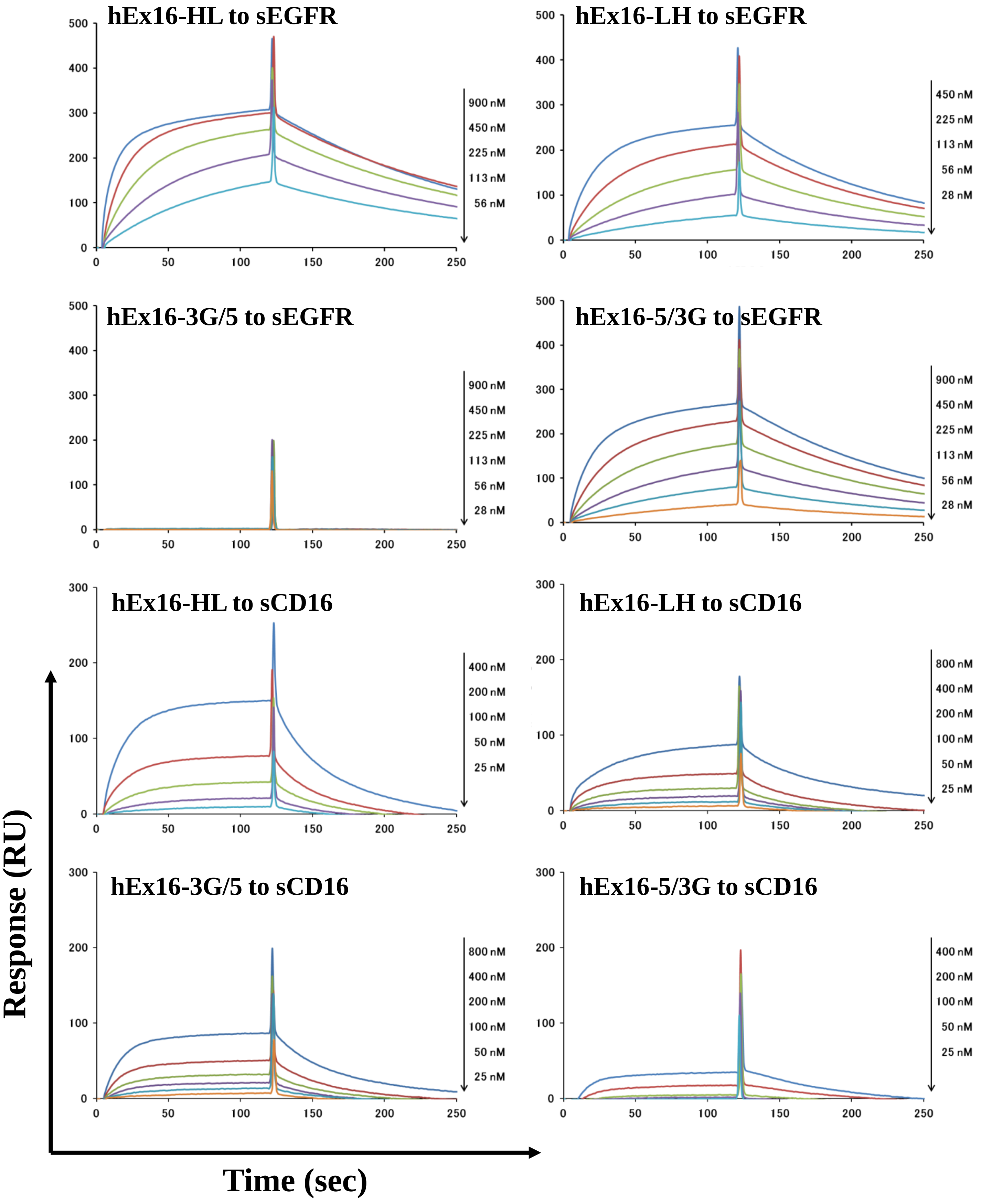

SPR sensorgrams for hEx16-Dbs. sEGFR or sCD16 was immobilized on the cells in a CM5 sensor chip up to 1,554 or 1,590 resonance units, respectively.

Figure S6

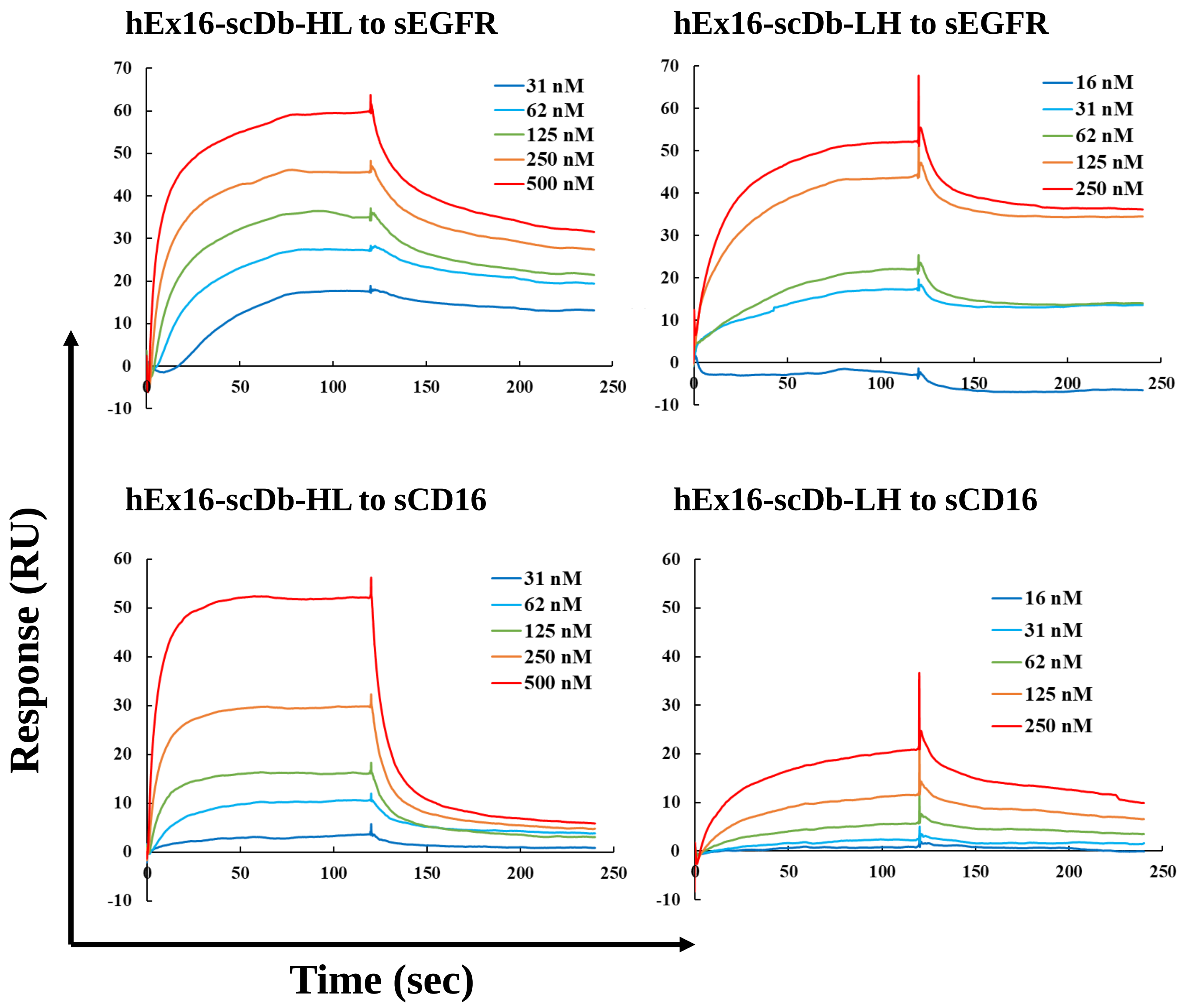

SPR sensorgrams for hEx16-scDbs. sEGFR or sCD16 was immobilized on the cells in a CM5 sensor chip up to 2,289 or 2,416 resonance units, respectively.
